# Activation of stimulus-specific processing regions at retrieval tracks the strength of relational memory

**DOI:** 10.3934/Neuroscience.2019.4.250

**Published:** 2019-10-17

**Authors:** Brion Woroch, Alex Konkel, Brian D. Gonsalves

**Affiliations:** 1 Department of Psychology, University of Illinois, Champaign, IL, USA; 2 Beckman Institute for Advanced Science and Technology, Urbana, IL, USA; 3 Department of Psychology, California State University, East Bay, Hayward, CA, USA

**Keywords:** relational memory, FMRI, recollection, memory strength, recapitulation

## Abstract

Many theories of episodic memory posit that the subjective experience of recollection may be driven by the activation of stimulus-specific cortical regions during memory retrieval. This study examined cortical activation during associative memory retrieval to identify brain regions that support confidence judgments of source memory in stimulus-specific ways. Adjectives were encoded with either a picture of a face or a scene. During a source memory test, the word was presented alone and the participant was asked if the word had been previously paired with a face or a scene. We identified brain regions that were selectively active when viewing pictures of scenes or faces with a separate localizer scan. We then identified brain regions that were differentially activated to words during the source memory test that had been previously paired with faces or scenes, masked by the localizer activations, and examined how those regions were modulated by the strength of the source memory. Bilateral amygdala activation tracked source memory confidence for faces, while parahippocampal cortex tracked source memory confidence for scenes. The magnitude of the activation of these domain-specific perceptual-processing brain regions during memory retrieval may contribute to the subjective strength of episodic recollection.

## 1. Introduction

Memory for episodes from one's past involves the coordination of multiple brain systems at the time of encoding and again during memory retrieval. These include cortical regions that process the components of an episode such as the people that were present, the time the event took place, or the setting in which the episode occurred. Structures in the medial temporal lobe (MTL), most notably the hippocampus, act to bind together these various elements of experience into a coherent episodic memory [1],[2]. This specific episodic memory can then be retrieved in response to a cue from one aspect of the memory, allowing inspection and re-experiencing of the entire original episode. Many models of episodic memory include the idea that such remembering involves the reactivation of the brain regions that were initially engaged at the time of encoding, and that this reactivation contributes to the subjective experience of remembering [3]–[5]. Empirical evidence derived from neuroimaging studies of memory retrieval have generally supported this idea, with several studies showing recollection-related reactivation during retrieval [6]–[10]. A potential implication of theories of recollection-related reactivation is that the strength or vividness of the subjective experience of recollection might be a function of the degree of reactivation of cortical regions, though this idea has not been extensively tested. The aim of the current study was to investigate perceptual brain regions that are activated during source memory retrieval and how this activation contributes to the remembering of different stimulus types, and how activation in these regions tracks the subjective strength of memory for specific stimulus types.

Some of the brain regions involved in episodic memory retrieval are engaged in a stimulus-general manner, and contribute to episodic memory regardless of the contents of the memory. This is true of the hippocampus, which participates in the binding and retrieval of the elements of episodic memory regardless of stimulus content. Many neural network models of episodic memory retrieval include the idea that one role of the hippocampus is to reactivate associated memory information through a process of pattern completion [11]–[14]. The advent of functional neuroimaging methods has provided ways to assess the assumptions of pattern completion and cortical reactivation. Studies have generally found that perceptual processing regions that represent the specific kind of associated information tend to be active during memory retrieval in response to a cue, even when the associated information type is not presented [7],[15]–[23]. For example, if a word had been encoded accompanied by a picture of an object, presenting the word alone at retrieval will often activate brain regions involved in the perception of object information. In addition to the reactivation of perceptual information, studies have also demonstrated effects associated with the reactivation of emotional associations [24],[25] as well as reactivation of cognitive or strategic processing engaged at encoding [10],[26],[27].

Several studies have taken the additional step of linking cortical reactivation effects with the process of recollection, which includes the retrieval of associated information, as opposed to familiarity, which is thought to reflect memory for just the item itself, with the idea that reactivation of associated information is a hallmark of recollection [10],[20],[21]. The general findings seem to confirm the notion that cortical reactivation is associated with recollection of episodic memory. However, there are fewer attempts to investigate how variations in these reactivations might be associated with subjective variations in the strength of recollection or with variations in objective accuracy or specificity of recollection. Evidence from the behavioral recognition memory modeling literature suggests a graded recollection process and a continuous variation in the memory strength of recollection [28]–[33]. Some studies have investigated the specificity of source retrieval [28],[29] and variations in the amount of retrieved information [8],[34],[35]. Previous research has focused on the role of ventral parietal cortex in the strength of recollection, showing that this region is modulated by differences in subjective ratings of the amount of information recollected [35],[36]. The precise function of this parietal region is unclear, though some have suggested that it may play a role in the orienting of attention to reactivated memory representations or may serve as an episodic buffer in working memory [34],[37]. Regardless of the exact function of this region, it appears to be stimulus-general, tracking subjective recollective memory strength regardless of the kind of information being retrieved.

The aim of this study was to investigate how cortical reactivation may vary with the subjective strength of source recollection. A few studies have attempted to address this question, and have found evidence that the degree of reactivation of stimulus-specific regions seems to scale with confidence in source memory decisions [38], which is often used as a proxy for underlying memory strength. Studies have also found that source misattributions are associated with reactivation of the incorrect memory content [39]. We sought to contribute additional evidence to these specific questions, taking advantage of the current understanding that distinct brain networks seem to be involved in processing faces and scenes. A network of regions including the superior temporal sulcus, amygdala, and fusiform cortex is more active in response to viewing faces than other types of stimuli [40]. In contrast, scene perception activates a network of brain regions involved in object perception, but one region known as the parahippocampal place area [41] seems to be most active to scene stimuli. These brain regions are also selectively activated during visual imagery of the respective stimulus type [42]. Furthermore, it has also been found that these stimulus-specific brain processing regions can be reactivated during associative memory tasks [10],[21].

We hypothesized that, as has been previously found, stimulus-specific cortical processing regions will be activated in response to associated memory cues during retrieval, including the face-specific and scene-specific regions listed above. Specifically, we expected that the magnitude of this cortical activation would be positively related to confidence in memory for the associated information that is not presented during retrieval, but is presumably reactivated in response to a previously associated cue. To test this, we showed participants words paired with faces or scenes during encoding. At retrieval, just the word was shown and subjects were asked if it had been paired with a face or scene. We expected that stimulus-specific processing regions, identified by fMRI localizer scans, would be activated in response to words that had been previously paired with that stimulus type. The magnitude of this activation was expected to increase as a function of source memory strength, indexed by subjective confidence in a source memory judgment. Thus the specific goal of the experiment was to show that subjective confidence in accurate source retrieval should be associated with corresponding increases in activity in stimulus-specific processing regions that represent the type of source information being retrieved.

## 2. Methods

### 2.1. Participants

Eighteen young adults participated in this study. The target N of 18 was determined based on a prior unpublished study that examined similar parametric modulations with source confidence. The data from 14 participants (6 F; ages 20–35 years old, *M* = 25.6) were included in the analysis. Data from 4 participants were excluded from analysis due to excessive motion within the MRI scanner (greater than 6mm in any one plane). By self-report, all participants were right-handed, had no history of psychiatric or neuropsychological disorders, and were not currently taking any psychotropic medications. All subjects gave written informed consent prior to participation in the study, which was approved by the University of Illinois Institutional Review Board, and were compensated $15/hr.

### 2.2. Procedure

The participants performed a recognition memory experiment with stimuli comprising of words, pictures of faces, and pictures of “places” (photographs of scenes), as well as a 1-back localizer task with just pictures. The words were 754 adjectives between 4 and 10 letters in length (M = 7.3). The face stimuli were comprised of 300 male and 300 female color photographs faces selected from a face database used in a previous study [43]. There were 720 color photographs of scenes, equally represented by 6 categories (mountains, beaches, cities, forests, highways, and industry) used previously by Walther et al. [44]. The stimuli were randomly assigned to the practice, memory experiment, and localizer task for each participant, with the condition that there were equal numbers of scenes from each category in each task as well as equal numbers of male and female faces.

**Figure 1.**
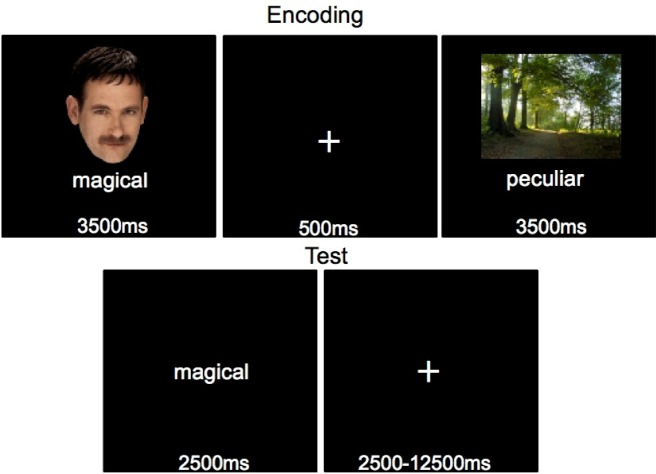
A sample trial from the source memory test. Pairs of words and pictures were shown at encoding. During the test a word was shown and participants responded with the type of stimulus, face or place.

The memory task consisted of a Study phase, which took place outside the MRI scanner, and a Test phase, which was performed within the MRI scanner. Prior to the experiment, participants completed a practice version of the entire memory experiment (encoding and source test), consisting of 48 stimuli not used in the remaining experiment. During the Study phase, participants studied 504 words randomly presented one at a time beneath a picture of either a face or a scene for 3500ms each, with a 500ms ITI (Figure 1). While the stimuli were on the screen, the participant rated how well the word described the picture on a 6-point scale. The first and last 2 stimulus pairs were not tested, to mitigate the effects of primacy and recency. The participants then entered the MRI scanner to perform the Test Phase, with approximately 30 minutes elapsing between the end of the Study phase and the beginning of the Test phase. The Test phase consisted of 500 words that had been previously studied (half with a face, half with a place), and 100 new words that had not been studied at encoding. During the memory test each word was centrally presented for 2500 ms and participants were instructed to imagine the image that had been previously paired with the word. They responded if the word was previously paired with a face or a place image and simultaneously rated their confidence on a scale from one to six (sure face, think face, guess face, guess place, think place, sure place). The hands that subjects used to make these six responses were counterbalanced across participants. Participants were instructed to guess face or place if they saw a word they did not remember seeing before. An additional 167.5 seconds of null fixation was randomly interspersed between trials, in 2.5–12.5 second intervals, throughout each block of the test to allow for deconvolution of the hemodynamic response to individual trial types. The test phase was broken into 6 blocks of 100 randomly selected trials. After each block participants were shown how many times they responded sure, think, or guess, regardless of stimulus type or accuracy, and encouraged to use the entire range of the rating scale.

Following completion of the memory task, participants performed a Localizer task with pictures of faces and places designed to identify face and place specific processing regions of the brain [44]. To ensure attention to the pictures, participants performed a 1-back test and pressed a button whenever a stimulus was immediately repeated. The pictures used in the localizer task were randomly chosen from the set of stimuli not used in the memory task. Each picture was shown for 750 ms with a 250 ms ITI. Stimuli were presented in 8 blocks. Each block consisted of either 12 faces or places, followed by 12 of the other stimulus type, followed by 15 seconds of fixation. A randomly selected 9 stimuli in each block were shown once and 3 were shown twice. The order of the blocks was counterbalanced within subjects so that each block had an equal chance of beginning with faces or places and an equal chance of being followed by a block of the same order.

### 2.3. fMRI Data Acquisition

Scanning was done using a 3T Siemens Allegra MRI with a 16-channel whole-head coil. After a T2-weighted anatomical scan, functional images were acquired using a gradient-echo echo-planar pulse sequence (TR = 2.5 s, TE = 25 ms, 44 interleaved oblique-coronal slices, 3.4 × 3.4 × 3 mm voxels, no gap, flip angle = 90 degrees, FOV = 220 mm, 274 volumes per run for 6 runs, followed by 152 volumes for one run). Oblique-coronal slice acquisition perpendicular to the main axis of the hippocampus was used to minimize susceptibility artifacts in the MTL during fMRI data acquisition. Slices were positioned to ensure complete coverage of the occipital lobe, at the expense of excluding the frontal poles for participants for whom whole-brain coverage was not possible. High-resolution T1-weighted MPRAGE anatomical images (1 mm isotropic voxels) were collected after the 6 experimental runs and 1 localizer run. Head motion was restricted using foam inserts. Visual stimuli were projected onto a screen behind the subject and viewed through a mirror mounted on the head coil. Responses were made with handheld button boxes.

### 2.4. fMRI data analysis

Data analyses were performed with Statistical Parametric Mapping (SPM5; Wellcome Department of Cognitive Neurology, London, UK, www.fil.ion.ucl.ac.uk) implemented in MATLAB 7.9 (The Mathworks Inc., USA). For each participant, functional images were adjusted for interleaved slice acquisition time and then subjected to affine motion correction. T2-weighted anatomical images were co-registered to the mean EPI volume across experimental runs, and high-resolution T1 MPRAGE images were co-registered to the T2-weighted images. The localizer scan EPI volumes were co-registered to the mean EPI image from the memory runs. All functional images were then normalized to the standard template based on the 152-subject MNI reference brain, resampled to 3 × 3 × 3 mm voxels, and smoothed with an 8mm FWHM Gaussian kernel.

The memory data were concatenated across runs and modeled using a general linear model. Event-related fMRI time-series data were convolved with the canonical hemodynamic response function, which was time-locked to the onset of each word. These functions were then used as covariates in the general linear model, along with time-derivative basis functions for each condition, regressors for global changes both within and between blocks, and regressors of the motion-correction parameters. A parametric modulator based upon behavioral source confidence was included to identify brain activity that increased with increasing confidence (low-medium-high). Only trials with a correct source memory decision were used for these analyses, such that we could look for variations in activity with variations in source confidence, for accurate source memory only. Least-square parameter estimates of the peak of the hemodynamic response function for correct face and place responses were calculated and adjusted by their respective parametric modulators. The parametric modulators identify regions that increase in activation linearly across the three confidence levels, regardless of where baseline activation falls among the three conditions. The average number of trials contributing to the analyses for low, medium, and high confidence source hits was 32, 55, and 56 for faces and 35, 56, and 46 for places. The source strength effects were then submitted to one-sample *t* tests at the group level, treating subject as a random effect, and masked by the localizer contrast. These analyses are reported at a threshold of *p* < 0.05, false-discovery rate corrected, with a 5-contiguous-voxel extent threshold. The localizer data was pre-processed in an identical way, and analyzed using a blocked design, contrasting blocks of faces and places. The data from each block was convolved with the canonical HRF. At the group level faces and places were contrasted against one another. The masks for the memory analysis were generated at a statistical threshold of *p* < 0.05, false-discovery rate corrected, with a 5-contiguous-voxel extent threshold.

In order to fully characterize the patterns of activations at each level of source memory confidence, percent signal change analyses were performed using MarsBar 0.41 for SPM5 [45]. Regions defined by the parametric memory strength analyses were submitted to a finite impulse response time-series analysis for the first eight repetition times (20 sec). The percent BOLD signal change was calculated for each strength level separately for each ROI.

## 3. Results

### 3.1. Behavioral results

**Figure 2.**
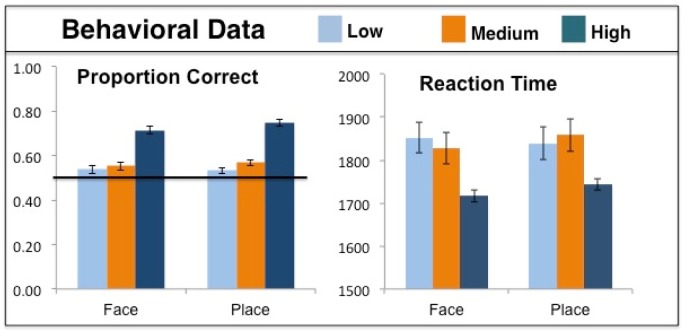
The behavioral data from the source memory test for low, medium, and high confidence responses for faces and places, with standard error bars. The black line at 50% correct represents chance performance.

The behavioral data were analyzed using a 2 × 3 (Face/Place × Confidence) repeated-measures ANOVA and are shown in Figure 2. Source memory accuracy in terms of percent correct was higher for places than faces overall [*F*(1,13) = 4.89, *p* = 0.046]. However, planned comparisons at each level of confidence individually revealed no difference in accuracy between faces and places [all *t*(13) < 1.48, all *p* > 0.161]. There was a main effect of response confidence on accuracy; as confidence increased so did behavioral accuracy [*F*(2,26) = 39.71, *p* < 0.001]. There was no interaction of response confidence and stimulus type [*F*(2,26) = 0.67, *p* = 0.521]. While response times decreased with increasing confidence [*F*(2,26) = 16.41, *p* < 0.001], there was no significant difference in response time between faces and places [*F*(1,13) = 1.24, *p* = 0.286], and no interaction of stimulus condition and response confidence [*F*(2,26) = 1.23, *p* = 0.309]. The accuracy for low confidence (guess) trials was not significantly above chance for either faces [54% vs. 50%, *t*(13) = 2.02, *p* = 0.064] or places [53% vs. 50%, *t*(13) = 1.38, *p* = 0.192]. However, accuracy for medium confidence (think) trials was above chance for both faces [55% vs. 50%, *t*(13) = 3.96, *p* = 0.002] and places [57% vs. 50%, *t*(13) = 5.518, *p* < 0.001]. The proportion of total responses that participants allocated to high, medium and low confidence face judgments and high, medium, and low confidence scene judgments are shown for Face, Place, and New stimuli in Table 1.

**Table 1.** Mean proportion of responses to each stimulus type falling into the six confidence response bins.

	**Stimulus Type**		
**Response**	**Face**	**Place**	**New**
High Confidence Face	0.22	0.10	0.09
Medium Confidence Face	0.22	0.18	0.18
Low Confidence Face	0.13	0.12	0.19
Low Confidence Place	0.13	0.14	0.20
Medium Confidence Place	0.18	0.23	0.22
High Confidence Place	0.07	0.18	0.07

### 3.2. fMRI results

The analysis involved two stages. First, we located stimulus-specific brain activations, face or place, using a localizer task. The activations identified in the localizer scans are listed in Table 2. Second, we identified brain regions more active during the source memory task, when cued with words alone, depending on which stimulus type was associated with the word during encoding (places or faces), and increased in activity with increasing behavioral confidence. Those regions were masked to only include regions from the localizer scan. We then extracted the percent signal change from the regions of overlap between the localizer activations and the memory confidence activations, to look at the activity level for each confidence level individually.

**Figure 3.**
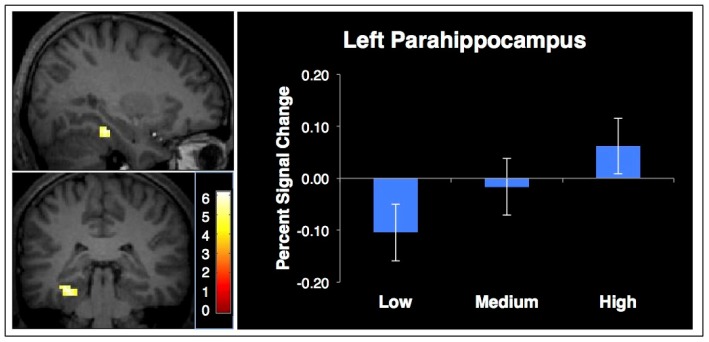
Brain activation that increases with confidence for words associated with places (left: x = −30 y = −33 z = −18), masked by regions from a place-face localizer. The graph shows the percent signal change of the parahippocampal activation to the associated word at each level of confidence with within-subject error bars.

The initial comparison looked for brain regions more active for places than faces that increased as a function of source memory confidence. A contrast of places minus faces from the localizer task revealed brain regions more active when viewing places, which was used as a mask for the memory test data. We then analyzed correct place trials from the source memory experiment, and identified regions that were parametrically modulated by increasing mnemonic confidence. The only region with statistically significant activation was the left parahippocampal cortex. The percent signal change from this region (peak at x = −30, y = −33, z = −18, *Z* = 4.14, 21 voxels) is plotted in Figure 3 for each level of confidence to show the amount of activation in each condition separately.

**Figure 4.**
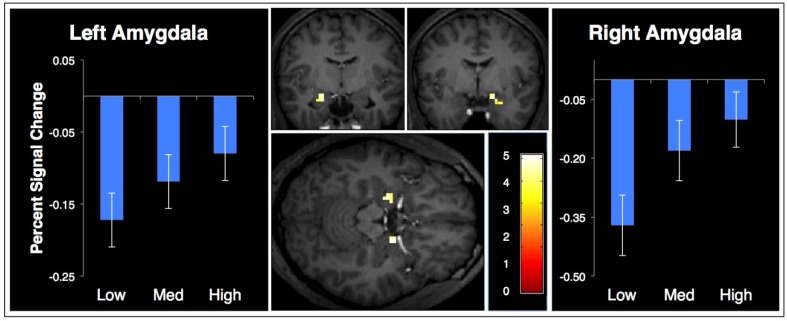
Brain activation that increases with confidence for words associated with faces (x = −21, y = −9, z = −15), masked by face-place activation from a localizer. The graphs show the percent signal change of the left and right amygdala to a word at each level of confidence with within-subject error bars.

The same strategy was employed to look for confidence-sensitive face processing regions. A contrast of faces minus places during the localizer task revealed brain regions more active when viewing faces than places. These activations were used as a mask for activity from the memory test. We assessed activation from correct face memory trials that parametrically increased activity with increasing mnemonic confidence. The only regions from this analysis were the bilateral amygdalae, shown in Figure 4. The percent signal change from these regions (left peak: x = −21, y = −9, z = −15, *Z* = 3.42, 16 voxels; right peak: x = 18, y = −3, z = −15, *Z* = 3.58, 12 voxels) is plotted separately for each level of response confidence.

Table 2.Regions activated in the localizer scans, with a false discovery-rate (FDR) corrected *p* < 0.05, and a 5 voxel cluster threshold.

**Table:** A: Face > Place

Hemisphere	Region	x	y	z
R	Middle Temporal	51	−60	0
L	Amygdala	−21	−9	−15
R	Superior Temporal	57	−42	15
R	Amygdala	15	−6	−12
R	Fusiform	45	−54	−18

**Table:** B: Place > Face

Hemisphere	Region	x	y	z
L	Parahippocampal Cortex	−27	−39	−15
R	Parahippocampal Cortex	21	−30	−18
L	Middle Occipital	−39	−87	21
R	Middle Occipital	33	−81	24
L	Middle Occipital	−21	−99	3
L	Superior Parietal	−21	−63	54

To reveal brain regions that increase or decrease in activity as a function of memory strength in a stimulus-general way, independent of the source information being retrieved, we performed a contrast of all correct memory trials, including both faces and places, parametrically modulated by increasing memory strength. The brain regions that were increasingly and decreasingly active are summarized in Table 3.

Table 3.Brain regions that are increasingly/decreasingly active with increasing stimulus-general memory strength.

**Table:** A: Increase

Hemisphere	Region	Voxels	x	y	z	Z-score
MTL						
L	Perirhinal Cortex	29	−27	−12	−33	4.06
L	Parahippocampus	28	−21	−39	−6	3.89
R	Hippocampus	9	27	−24	−15	3.86
Other						
L	Superior Medial Frontal	130	−6	30	45	4.34
L	Insula	169	−30	21	−6	4.31
L	Caudate	172	−15	0	21	4.16
R	Caudate	98	12	6	18	3.68
L	Precuneus	97	−12	−69	33	3.65

**Table:** B: Decrease

Hemisphere	Region	Voxels	x	y	z	Z-score
R	Supramarginal	61	63	−9	30	4.17
R	Precuneus	30	9	57	54	4.05
R	Angular	15	30	66	45	3.96
R	Middle Frontal	8	45	48	12	3.88
R	Middle Frontal	6	24	57	27	3.36

We also hypothesized that activation of stimulus-specific regions may account for incorrect responses and how the participant responds to novel words. We examined trials in which the participant responded to the face/place question incorrectly as well as responses to novel words. We performed two contrasts, comparing incorrect trials for faces with the incorrect trials of places, and face or place responses to novel words, again masked by regions from the localizer scan. There were not enough trials to look at each level of response confidence separately, so low, medium, and high confidence were collapsed together for these analyses. We used a more liberal statistical threshold for this more exploratory analysis due to low numbers of trials (*p* < 0.001, uncorrected, with a 5-contiguous-voxel extent). We found no significant activation for words paired with a place during encoding and incorrectly endorsed as having been paired with a face during the source memory task or for novel words judged as having been paired with a face. However, words paired with a face at encoding but incorrectly endorsed as having been paired with a place during the source memory test produced activation in the left parahippocampus (x = −33 y = −27 z = −15, Z = 4.59, 15 voxels). New words for which participants chose “place” as the source also elicited parahippocampal activation (x = −30 y = −48 z = −6, Z = 3.53, 7 voxels).

## 4. Discussion

This study examined cortical activation during associative memory retrieval to identify brain regions that support confidence judgments of source memory in stimulus-general and stimulus-specific ways. We identified stimulus-specific processing regions using a separate localizer task. We hypothesized that the magnitude of activation of stimulus-specific processing brain regions during a source memory task would scale with confidence for that specific kind of information. Thus we expected that regions more active for faces or places during our localizer would be increasingly active with increasingly confident source memory of the respective stimulus type. We identified brain regions that were more active during correct source memory that increased their activity parametrically with source memory confidence, masked by regions that differed between activations from a face or place localizer task. We also examined brain regions whose activity increased with source memory strength, as measured by confidence, in a stimulus-general way, independent of associated stimulus type. Finally, we examined the brain response to novel and incorrect words.

Localizer scans for scene stimuli identified several occipital and temporal regions, including several clusters in the parahippocampal cortex that contribute to scene (place) processing. During the source memory task, when participants were correct in remembering that a word had been previously paired with a scene, the parahippocampal cortex increased in activity with increasing behavioral confidence. This parahippocampal region was within the regions activated in the scene localizer task. The parahippocampal regions observed in the current study included regions characterized previously as the parahippocampal place area [41] as well as more anterior regions. It should also be noted that a nearby area of parahippocampal cortex was activated in the contrast that identified stimulus-general reactivation effects. Parahippocampal cortex is thought to be involved in the representation of context (which naturally includes places), such that the reactivations found in the stimulus-general contrast may reflect reactivation of other aspects of context than what we presented, though this explanation is necessarily speculative.

The activation of this parahippocampal region may also be related to guessing behavior or false source memory on the memory test. The parahippocampal cortex was active for incorrect place source memory, words incorrectly judged to have been paired with a place during the memory test but were originally paired with a face during encoding. The parahippocampal cortex was also active for novel words judged to have been paired with a place. The study design did not yield enough trials to look at the contribution of confidence to this effect, so it is unclear how the magnitude of this response relates to confidence. We found no evidence of brain regions that contribute in a similar way to incorrect face source memory, words incorrectly judged to have been paired with a face during the memory test but were actually unstudied or paired with a place during encoding. This is generally consistent with the idea that increases in activation of stimulus-specific regions is more strongly associated with accurate source memory retrieval, supporting the interpretation that these effects reflect the retrieval of face or place information that was encoded at study. However, the analyses of inaccurate source memory and novel word responses were noisier due to smaller numbers of trials, so care is required in interpreting these null effects. A study specifically designed to examine brain regions that contribute to incorrect source judgments is needed to draw conclusions about the functional significance of these observations and to observe how this activity might interact with confidence.

The face localizer task revealed activity in several brain regions previously associated with face processing: the right fusiform cortex, right superior temporal sulcus, and bilateral amygdalae [40]. During retrieval, when participants viewed a word that had been studied with a face, and correctly identified it as such, regions in bilateral amygdala increased in activation with increasing confidence in the “face” source memory decision (Figure 4).

Given the amygdala's role in processing emotion, we were concerned that this observed activation reflected the possibility that the words more likely correct in the face condition may have been more emotionally arousing than those in the place condition. Thus, we identified words that were more likely to be associated with accurate face source memory than place across subjects. We obtained the mean arousal ratings for most of the words used in this experiment from a normed database [46]. We found no difference in the emotional arousal ratings between words associated with accurate face source memory compared to accurate scene source memory [*t*(355) = 0.66, *p* = 0.438]. Although we did not collect arousal ratings from the participants in the current study, this provides some evidence against the idea that amygdala activity observed in the current study was due to the emotional content of the words themselves. Our conclusion is that the amygdala activity observed is related to source memory specific to face processing.

We expected that activation of regions typically associated with face processing, including fusiform face area (FFA) and superior temporal sulcus (STS), would be associated with face source memory confidence. However, those regions were not significantly more active during face source memory than during scene source memory in our parametric analysis. We followed up our parametric analysis by conducting region of interest analyses of these face-processing regions. There was no evidence of stimulus-specific activation that increased with source memory confidence in either the FFA or STS. It is unclear why these regions, well known to be involved in face processing, did not also show memory-related reactivation effects. It may be that top-down reactivation of face memory does not include these regions, or that these regions are reactivated, but not in a way that scales with confidence in source memory. Another potential explanation is that the sample size used in this study may have been insufficient to detect these potentially weaker effects. Further study is needed to address these potential explanations.

This study contributes data that supports the idea that activation of stimulus-specific processing regions of the brain support recollection of associated source information. The activation of different brain regions during recollection is related to the source memory decision, and increasing activation of these regions scales with increasing confidence. The activation of these regions during source memory may be related to false memory of specific stimulus types, although follow-up tests are needed. Regions of the medial temporal lobe, including the hippocampus, parahippocampal cortex, and perirhinal cortex all increased their activation with increasing source memory confidence in a stimulus-general way, regardless of the associate stimulus type. These regions have been previously associated with memory strength [47]–[49].

A remaining question of interest is, what type of information is being represented by this cortical activation? Based on the involvement of these regions in visual imagery, we conclude that this activity reflects the recollection of the stimulus that had been studied with the word. However, an equally plausible explanation is that the activation represents access of category-level information since that is the only information diagnostic for the source memory decision. Brain regions representing “scene” information that activate to associated cues may reflect the recollection of the exact scene stimulus that was initially studied. Or, it may reflect basic category-level information, such as the spatial information diagnostic of a scene compared to a face, or scene category level information, such as a forest compared to a mountain. Future experiments will need to discriminate these potential interpretations.
